# Diacetin, a reliable cue and private communication channel in a specialized pollination system

**DOI:** 10.1038/srep12779

**Published:** 2015-08-06

**Authors:** Irmgard Schäffler, Kim E. Steiner, Mark Haid, Sander S. van Berkel, Günter Gerlach, Steven D. Johnson, Ludger Wessjohann, Stefan Dötterl

**Affiliations:** 1 Department of Ecology and Evolution, University of Salzburg, Hellbrunnerstr. 34, 5020 Salzburg, Austria; 2 Department of Plant Systematics, University of Bayreuth, 95440 Bayreuth; 3 School of Life Sciences, University of KwaZulu-Natal, Scottsville, Pietermaritzburg, South Africa; 4 Leibniz Institute of Plant Biochemistry, Weinberg 3, 06120 Halle (Saale), Germany; 5 Botanical Garden München-Nymphenburg, Menzinger Str. 65, 80638 München, Germany

## Abstract

The interaction between floral oil secreting plants and oil-collecting bees is one of the most specialized of all pollination mutualisms. Yet, the specific stimuli used by the bees to locate their host flowers have remained elusive. This study identifies diacetin, a volatile acetylated glycerol, as a floral signal compound shared by unrelated oil plants from around the globe. Electrophysiological measurements of antennae and behavioural assays identified diacetin as the key volatile used by oil-collecting bees to locate their host flowers. Furthermore, electrophysiological measurements indicate that only oil-collecting bees are capable of detecting diacetin. The structural and obvious biosynthetic similarity between diacetin and associated floral oils make it a reliable cue for oil-collecting bees. It is easily perceived by oil bees, but can’t be detected by other potential pollinators. Therefore, diacetin represents the first demonstrated private communication channel in a pollination system.

Most angiosperms are pollinated by a diverse subset of all potential flower-visiting animals^1^. However, some plants exhibit extreme specialization for pollination in that only one or a few animal species belonging to a single functional group (e.g. bees, butterflies, or birds) act as their pollinators^2^,^3^. Benefits to plants that are highly specialized for pollination include reduction of pollen loss and clogging of stigmas with foreign pollen, and a decrease in interspecific gene flow, especially if the pollinators show fidelity and are equally specialized in the choice of their host plants^2^,^4^. Advantages of being a specialist pollinator are a higher foraging efficiency, potentially reduced interspecific competition from other pollinators, and the possibility of evolving reciprocal adaptations for exploitation of particular host plants^4^,^5^. Examples of specialized pollination systems include the interactions between figs and fig wasp pollinators, between long-spurred flowers and their long-tongued fly or moth pollinators, and between oil secreting plants and their oil-collecting bee pollinators^2^,^4^. In many specialized pollination systems, floral scent is the most important floral signal for pollinator attraction and this allows recognition of the host by the pollinator^6^, however, other modalities, such as visual cues, are also typically involved in pollinator attraction^7^. Scent-mediated specificity in pollinator attraction has been suggested to occur through either one of two mechanisms: 1) the production of unique compounds or 2) the production of specific blends of common compounds. The first type can be viewed as a sensory ‘private channel’ between the plant and its intended pollinator if the critical scent components are easily detected by the intended receivers (i.e., pollinators) while remaining undetected by unintended receivers^6^. These two alternative mechanisms (unique compounds vs. blends of common compounds) have been variously implicated in case studies of specialized pollination systems. For example, sexually deceptive orchids mimic female sex pheromones of various Hymenoptera by emitting either uncommon compounds (e.g., chiloglottone^8^, pyrazines^9^), or blends of commonly occurring hydrocarbons such as alkenes or alkanes^10^ to attract pollinating males that are searching for females. Although unusual or unique compounds are good candidates for private communication channels, the assumption that these are readily detected by pollinators and undetected by other potential flower visitors has not been tested previously in any pollination system where private channels are assumed to operate^6^. In (sexually) deceptive systems mediated by uncommon compounds, the plants exploit existing olfactory capabilities and preferences of specific pollinators. In non-deceptive, reward-based pollination systems, the olfactory capability for detecting uncommon or unique compound(s) may be the result of an adaptation in the olfactory circuitry (receptors, binding proteins, neurons) that evolved to recognize the specific food plant(s). Although such adaptations to specific scent compounds of non-deceptive host plants have not been demonstrated in any pollinator, it is known that different insects detect and respond differently to specific compounds from their habitat. This variability in the periphery of the olfactory circuitry of insects demonstrates the evolutionary potential for divergence in response to scent components, even among insects that are closely related^11^,^12^,^13^.

The highly specialized pollination mutualism between floral oil secreting plants (henceforth oil plants) and oil-collecting bees (henceforth oil bees) has evolved in more than ten plant families and two families of bees^14^,^15^,^16^,^17^. Plant species which produce and secrete floral fatty oils, (mostly) in lieu of nectar, occur throughout the globe in Neotropical, Palaeotropical, Afrotemperate, and Holarctic floristic regions^18^. In each area, this oil is collected by females of only a few specialized oil bee species and these are either members of the Apidae (Palaeotropical and Neotropical regions) or Melittidae (Holarctic and Afrotemperate regions). The oil is used by these bees as larval food provisions (e. g.^19^) and as a constituent of the cell lining within the nest^19^,^20^. The function of this cell lining is to protect the larval provision and the immature stages from water and pathogens, such as fungi^15^. The use of floral oils in nest cell lining is exceptional in bees, and only found in oil-collecting bees, and not in other bees, which usually use secretions of the large Dufour’s gland for the cell lining^21^. In *Macropis* and other oil bees, the Dufour’s gland is small and strongly reduced^20^,^22^. In bees, oil collection has evolved at least seven times, and, in plants, oil as a floral reward has developed independently at least 28 times^18^. Despite the widespread nature and repeated evolution of this pollination system, it is generally rather rare compared to nectar- or pollen-based reward systems^18^, involving approximately 1700 species of plants and 370 species of bees.

Most interactions between oil plants and oil bees are obligate mutualisms. The oil bees are the sole pollinators of the plants and the oil plants are essential hosts for the bees^23^,^24^. Although some oil plants are also visited to a small extent by non-oil bees, such bees play only a minor role as pollinators^24^. As oil plants and oil bees are dependent on each other, an effective communication system can be expected to have (co)evolved in this pollination system. As in other specialized pollination systems, floral scent is important for the interaction between oil plants and their bee pollinators. Behavioural experiments with naive females of the European *Macropis fulvipes* with no prior *Lysimachia* experience revealed that olfactory cues of their host plant, *Lysimachia punctata,* are more important for host location than visual cues. This use of olfactory cues for locating hosts seems to have a genetic basis, as scent-based attraction of *M. fulvipes* bees to *Lysimachia* flowers was not dependent on their previous foraging experiences^7^,^25^.

Compounds responsible for attracting *M. fulvipes* are present in solvent extracts of complete flowers and in extracts of the floral oils^25^ (Schäffler unpublished). Both complete flowers and floral oils release a wide variety of compounds^25^, however, the specific compound(s) eliciting the behavioural response in oil bees have remained unknown. One uncommon compound, 1-hydroxy-1-phenyl-2-propanone, present in both flower and oil samples has been suggested to play a role in *Macropis* bees attraction^6^,^25^, however, it has not been found to attract bees in behavioural tests^26^.

The finding that solvent extracts of oil are capable of attracting *Macropis* bees led us to speculate that the floral oils or compounds involved in the biosynthesis of these oils are involved in pollinator attraction. Such compounds would be an ideal signal for *Macropis* to locate oils, because it would directly indicate the presence of oils (i.e., be an honest signal sensu Raguso^27^). In animal communication terminology, the biosynthetic link between such a compound and the reward would make the compound an “index signal”^28^,^29^. Interestingly, oil plants around the world all produce quite similar oils. These consist typically of mono-, di-, or triacetylated glycerols or free fatty acids (typical chain lengths: C_16_, C_18_) with a hydroxyl or acetyloxy group on the beta carbon^14^,^15^,^16^,^30^,^31^. Thus, oil flowers around the world may advertise their oil rewards with a similar signal. Generally, in most pollination systems, pollinators locate rewards using volatile signals derived from biosynthetic pathways (e.g. terpenoids, aromatics, aliphatics) that are not directly linked to production of rewards (e.g. sugars and/or protein)^32^. Such “conventional signals”^28^, however, can also honestly indicate a reward^33^. Pollination systems in which the signal itself is the reward or that are based on compounds that are biosynthetically very similar to the rewards are very rare, but can be found in systems involving male perfume collecting euglossine bees^34^ and male tephritid flies^35^. Euglossine males use these compounds during courtship behaviour^36^, whereas male flies use them directly^37^ or after conversion into sex pheromones^38^, for mate attraction.

Based on (i) the similarity in chemical structure of floral oils among oil plants around the world, (ii) the observation that floral scent extracted from oil attracts oil bees, and (iii) the fact that oil flowers are pollinated almost exclusively by oil bees, we address the hypothesis that the oil flower/oil bee pollination system is mediated by a volatile private communication channel which is derived from the pollinator reward (viz. oil).

## Results

### Detection of EAD-active compounds in oil flowers

**Figure 1:**
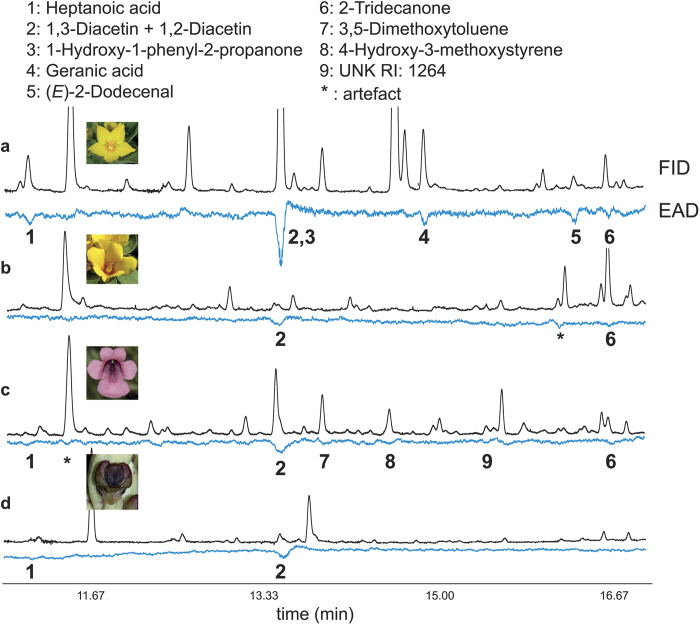
Electroantennographic responses of *Macropis fulvipes* oil bees to scents of different oil flowers. GC-FID (black line) and GC-EAD responses of *M. fulvipes* antennae (inverted blue line) to floral extracts of *L. punctata* (**a**), *L. congestiflora* (**b**), *Diascia integerrima* (**c**), and *Corycium dracomontanum* (**d**); (UNK RI 1264: unknown compound, kovats retention index 1264; m/z: 122, 78, 106, 51, 50). Plant photographs by Irmgard Schäffler (**a**,**b**) and Kim E. Steiner (**c**,**d**).

In the GC-EAD analyses with antennae of *M. fulvipes* and scent samples collected from four different oil plants, we found only one EAD-active compound, diacetin, that occurred in all of these plant species (Fig. 1). Two EAD-active compounds (heptanoic acid, 2-tridecanone) occurred in three of the plant species, whereas six compounds (1-hydroxy-1-phenyl-2-propanone, triacetin, (*E*)-2-dodecenal, 3,5-dimethoxytoluene, 4-hydroxy-3-methoxystyrene, UNK RI: 1264) occurred in one of the four plant species.

### Occurrence of EAD-active compounds

The most widespread EAD-active compound was diacetin, which occurred in 41 of the 50 (82%) studied oil species, but in only one of the eight (12.5%) related non-oil species examined. It was present in all of the Holarctic (seven) and South African (18) oil species, as well as in 16 (73%) of the Neotropical oil species (Table 1, for complete list see Table S1). Nearly as widespread as diacetin was 2-tridecanone, which was found in 34 (68%) of the oil species and in one non-oil species. Heptanoic acid was detected in 20 (40%) and 4-hydroxy-3-methoxystyrene in 18 (36%) of the oil species, whereas the remaining EAD-active compounds occurred in less than 10 (20%) of the oil secreting species.

### EAG - antennal responses to diacetin among oil and non-oil bees

**Figure 2:**
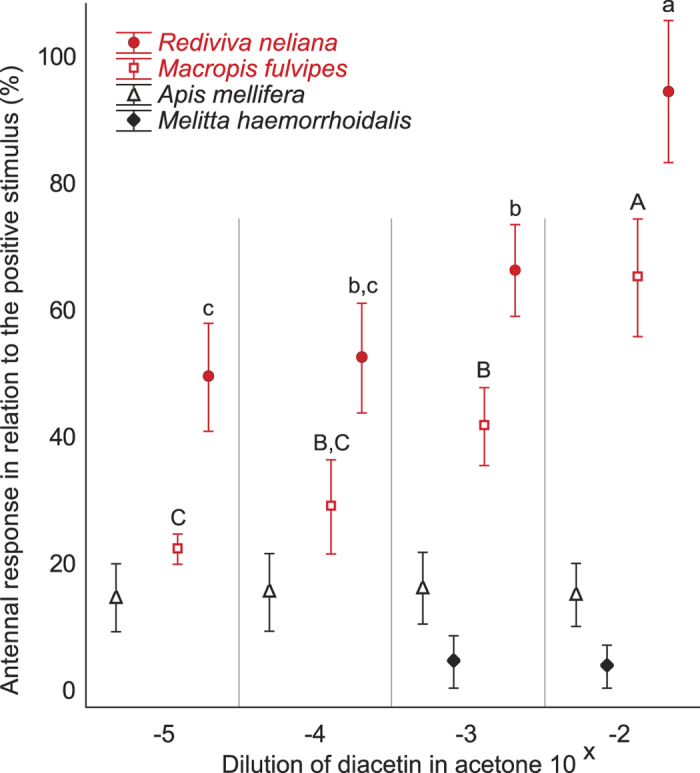
Physiological dose-response curves of different bees for diacetin. Electroantennographic responses (EAG) of oil bees (*Macropis fulvipes*/*Rediviva neliana*) and non-oil bees (*Melitta haemorrhoidalis*/*Apis mellifera*) to different dilutions of diacetin. SEM = standard error of the mean. Means (within the same species) that share same letters or do not have letters are not significantly different.

Overall analysis revealed significant effects of *bee species* (F_2,17_ = 15.97, *P* < 0.001), *dilution* (F_3,51_ = 60.21, *P* < 0.001), and the *bee species *× *dilution* interaction (F_6,51_ = 17.94, *P* < 0.001) on antennal responses to diacetin. Antennal responses increased with increasing concentration of diacetin for *M. fulvipes* and *Rediviva neliana* oil bees, but not for honey bees (Fig. 2). As was the case for honey bees, there was no *dilution* effect of diacetin for the non-oil bee *Melitta haemorrhoidalis* (t = 1.00, df = 4, *P* = 0.37).

Responses to the highest concentration of diacetin were stronger than to acetone in antennae of *M. fulvipes* (t = 6.32, df = 4, *P* < 0.01) and *R. neliana* (t = 9.44, df = 5, *P* < 0.001), but not in those of *M. haemorrhoidalis* (t-test: t = 1.00, df = 4, *P* = 0.37) and *A. mellifera* (t-test: t = 1.13, df = 8, *P* = 0.29).

### Behavioural experiments

**Figure 3:**
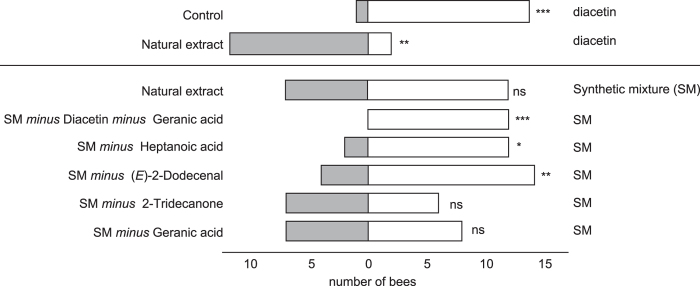
Behavioural experiments testing the attractiveness of floral compounds of *Lysimachia* oil flowers to *Macropis oil* bees. Approaches of naive *Macropis fulvipes* females to diacetin alone (against acetone as a control), natural floral extracts of *Lysimachia punctata*, and complete (diacetin and four other compounds) and partially depleted synthetic mixtures of EAD-active compounds identified in *L. punctata* floral extracts. Exact binomial test: ns: *P* > 0.05; **P < 0.01; ***P < 0.001).

In two-choice experiments conducted in the flight cage, diacetin alone attracted significantly more bees than did a negative control, but significantly less bees than did a natural floral extract (Fig. 3). However, the creation of a synthetic mixture with diacetin and four additional EAD-active compounds increased the attractiveness to the same level as the natural floral extract (see also supplementary data, Movie 1).

Bees responded differently to samples (natural extract, reduced synthetic mixtures) that were tested against the complete synthetic mixture (Fisher’s exact test: *P* = 0.01). Removal of geranic acid or 2-tridecanone from the mixture had no effect on attractiveness to *M. fulvipes* females, but removal of heptanoic acid or (*E*)-2-dodecenal reduced the attractiveness significantly relative to the complete synthetic mixture (Fig. 3). When diacetin was removed from the mixture (together with geranic acid, see material and methods) bees were attracted only to the complete mixture (Fig. 3).

## Discussion

Our data demonstrate that flowers of most of the studied oil species around the world emit the fatty acid derivative diacetin. This compound elicits strong antennal responses in oil bees from different floristic regions and continents, but it does not elicit antennal responses in related non-oil bees. This suggests an olfactory adaptation in oil bees to this uncommon compound. Diacetin is a key signal in the *Lysimachia*-*Macropis* pollination system, but other compounds can also add to the attractiveness of a scent blend. Overall, our data suggest that diacetin is a private communication channel and honest signal in the oil flower/oil bee pollination system.

Diacetin, only recently described as a floral compound^39^, occurs as a floral scent constituent in most (82%) of the oil plant species tested, regardless of floristic region (Holarctic, Neotropical, Afrotemperate region) or plant lineage (Asparagales, Malpighiales, Ericales, Lamiales). These findings strongly suggest, therefore, that the production of diacetin in oil flowers has evolved independently several times, in accordance with the independent evolution of oil secretion in these flowers^18^,^40^.

In contrast to the widespread occurrence of diacetin in oil plants, we did not find diacetin in related non-oil species with one exception. In the non-oil secreting *Lysimachia thyrsiflora*, the sister species of oil secreting and diacetin emitting *L. vulgaris*^40^, this compound was detected in flower extracts and more recently also in headspace samples. The presence of diacetin in this non-oil species may be the result of a recent switch away from pollination by oil bees yet with retention of the ability to produce small quantities of floral oil and diacetin due to relaxed selection against its production^41^,^42^.

Diacetin has not been found in dynamic headspace collections from several oil species^25^,^43^, even though we identified it in solvent extracts of flowers of these same species. This suggests that diacetin is present only in small and hard to detect amounts in floral headspace samples (see also below). Interestingly, diacetin has not been identified in studies focusing on the chemistry of the floral oils^15^,^31^. We attribute this to its smaller size and higher volatility compared to the target non-volatile oils, and a methodology that did not allow its detection. The amount of diacetin available in the samples was quite small compared to the oils and this small amount may have been lost in the process of evaporating the “oil samples” to dryness.

**Figure 4:**
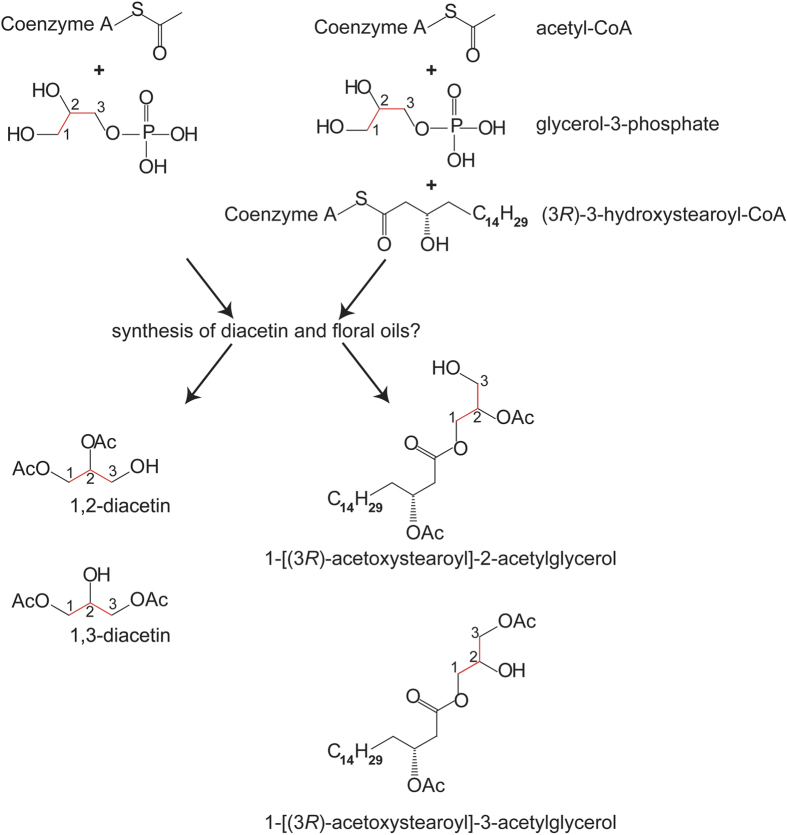
Diacetin is likely a derivative of the fatty floral oil biosynthesis. Schematic overview of proposed biosynthesis of diacetin and abundant floral oil components found in *Lysimachia punctata*.

The basic structure of floral oils (i.e. acylglycerols) is similar for the oil species found around the world and resembles that of the volatile compound diacetin as well as some lipids in plant tissues^31^. As exemplified by *L. punctata*, major compounds in the floral oil are 1-[(*3R*)-acetoxystearoyl]-2-acetylglycerol and 1-[(*3R*)-acetoxystearoyl]-3-acetylglycerol and both of these compounds are composed of a glycerol esterified with one acetic acid and with one substituted long-chain fatty acid (Fig. 4). Structural similarities of these two compounds with 1,2- and 1,3-diacetin are evident. It can be assumed that metabolic pathways or enzymes utilized, such as those involved in ester formation of glycerol with fatty acids^44^ (specifically 3-hydroxy/3-acetoxy fatty acids) or acetic acid, are to some extent identical for this group of lipids and for diacetin production (Fig. 4).

Since acetylation of glycerol or the backbone of the hydroxylated long chain fatty acids is almost universal in “non-volatile” floral oils^30^,^31^, it can be hypothesized that diacetin might be present in all oils of this type, whereas it may not be present in oils made up of other types of lipids (e.g. free fatty acids, classical triglycerides or wax esters, terpenoids). Indeed, we found diacetin in all plants having oils congruent with these criteria with the exception of *Momordica* (Cucurbitaceae) and *Bunchosia* (Malpighiaceae) species. Diacetin was also missing from *Nierembergia* (Solanaceae) species, but their oils do not consist of acetylated glycerols (Table S2). The common occurrence of diacetin with ‘acetylated’ floral oils supports the idea that these compounds are derived from the same metabolic pathway or, at least partially, rely on the same enzymatic endowment. Even if diacetin evolved initially as a by-product of oil synthesis and was subsequently co-opted by oil bees as an “index signal”^28^,^29^, it still represents a reliable cue for bees looking for floral oils. Parallels can be drawn to a communication system between male and female rattlebox moths (*Utetheisa ornatrix*). Here, the males use a volatile derivative of a larger defence compound, to indicate to the female the quality of the sequestered defence compounds that they pass along to the female during mating^45^.

Our data show that diacetin is widespread among oil species and a good candidate for use by oil bees around the world as a reliable cue for locating oil rewards. They also indicate that diacetin represents a private communication channel between oil plants and oil bees. In our electrophysiological measurements, diacetin elicited antennal responses in melittid bees from both Europe (*M. fulvipes*) and South Africa (*R. neliana*). It also elicits responses in another European *Macropis* species, *M. europaea* Warncke, and two additional South African *Rediviva* species, *R. brunnea* Whitehead & Steiner, and *R. pallidula* Whitehead & Steiner (Dötterl and Steiner, unpublished data). Diacetin did not, however, elicit significant antennal responses in the closely related non-oil melittid bee (*M. haemorrhoidalis*) or the honey bee (*A. mellifera*, Apidae). This difference in antennal response to diacetin between oil and non-oil bees demonstrates that the oil bees have specific olfactory adaptations in the periphery of the olfactory circuit to detect diacetin. This adaptation functions most likely at the level of the olfactory receptors or the olfactory binding proteins^11^,^46^,^47^, but additional adaptation in the brain (e.g. processing) cannot be excluded. Such adaptations towards volatile signals of host plants have not been described for any other pollinators and our next step will be to test whether oil bees belonging to the Apidae exhibit a similar positive response to diacetin.

Our bioassays with *M. fulvipes* and the EAD-active scent compounds of its host plant *L. punctata* point towards a key function of diacetin in host plant location. The presence of diacetin alone was sufficient to attract *Macropis* bees. Two other EAD-active compounds (heptanoic acid, (*E*)-2-dodecenal) were also behaviourally active. However, a mixture containing these two and two additional EAD-active compounds (2-tridecanone, geranic acid) but lacking diacetin did not attract bees when tested against a synthetic mixture that contained all compounds (Fig. 3).

Trace amounts of diacetin were found as a contaminant in our synthetic geranic acid sample and, therefore, we had to exclude geranic acid from our mixture in order to obtain a diacetin-free sample for the choice tests. These trace amounts proved sufficient to elicit behavioural responses in *Macropis*, because a synthetic mixture without diacetin but with geranic acid attracted *Macropis* bees (Schäffler, unpublished data). When removing only geranic acid from the complete synthetic mixture the bees did not discriminate between the depleted and the complete mixture, demonstrating not only that geranic acid has no influence on bee behaviour, but also that the absence of trace amounts of diacetin (when higher amounts are still present) did not influence the choice of bees. Overall, we conclude that diacetin and not geranic acid was responsible for the loss of attractiveness relative to the complete scent mixture when excluding both substances from the complete mixture. This confirms that diacetin is a key compound in attracting *Macropis*.

In addition to diacetin, heptanoic acid and (*E*)-2-dodecenal are used by *Macropis* bees for locating oil flowers. Heptanoic acid was detected in about 20 oil species in three floristic regions, and recently in a few oil and non-oil species^43^,^48^. The only other reported instance of a biological function for the compound is as a kairomone in an insect host-parasite communication system^49^. Even rarer is the floral scent compound (*E*)-2-dodecenal that was found in three floral oil secreting *Lysimachia* species. Until now, this compound was known from only a few South African oil orchids^43^ and from two species without floral oils^50^,^51^, and from a millipede where it acts as an insect deterrent^52^. In contrast, the EAD-active compound 2-tridecanone is very widespread among our oil species studied, among a large number of oil orchids of South Africa^43^, and among several non-oil species^48^,^50^,^51^, yet it did not influence the attractiveness of the synthetic mixture. This compound, known as a repellent for insects^53^ including generalized bee pollinators^54^, could act as a floral filter^39^,^43^ at least in the *Macropis*-*Lysimachia* pollination system to reduce visitation rates from inappropriate visitors that might remove pollen without providing adequate pollination.

Interestingly, while diacetin is very widespread among oil plants, the plants emit additional scent compounds, several of which are not widespread and do not occur in more than one or a few of the species studied^39^,^43^,^51^. There is a high overall variation in floral scent among oil plants, which is true for species within floristic regions and even for species pollinated by the same oil bee species (Holarctic:^39^, South Africa:^43^) as well as among floristic regions. These findings lead us to believe that diacetin is a reliable volatile marker for ‘non-volatile’ fatty oils throughout the world, whereas the emission of other compounds, like geranic acid, 3,5-dimethoxytoluene, or (*E*)-2-dodecenal, may be important for allowing bees to discriminate among co-blooming species. Scents distinguishable among plant species are known to promote effective pollen transfer within species and species integrity through flower constancy of pollinators^55^. However, we did not find diacetin in all of the floral oil species suggesting that they may emit diacetin in amounts too low for detection or that diacetin is not used as a signal by these plants. If the latter is true, compounds other than diacetin may occasionally be important as pollinator attractants. Since oil production has evolved independently in several families and some plants produce floral oils structurally dissimilar to diacetin^31^, it would not be altogether surprising if some oil species used different signals.

## Conclusion

Diacetin occurs in several floral oil plants around the world and is detected by oil bees from at least two continents. It allows the Holarctic *Macropis* oil bee, and probably other oil bees, to rapidly and efficiently locate their oil secreting host plants. Our data for *Macropis* and *Lysimachia* suggest that diacetin represents the first demonstrated private communication channel between a pollinator and its host plant. Notably, diacetin satisfies the two requirements of a private communication channel: 1) it is an uncommon compound and 2) it can be detected by its specialized and specific pollinators, but apparently not by other potential pollinators in the environment. We cannot rule out the possibility that one or more of the thousands of non-oil bees that we didn’t test may be able to detect diacetin, yet, there seems little selective value in evolving or retaining such an ability outside of an oil flower/oil bee relationship. Dated phylogenies show that *Lysimachia* and *Macropis* are of similar age making it plausible that they coevolved from the onset^18^. Thus, the described fine-tuned adaptation towards diacetin in the sensory apparatus of the bees and the chemical profile of the host plants may be the result of coevolutionary processes. The obvious sharing of the biosynthetic production by diacetin and floral oils, at least those with acetylation, make diacetin an ideal and reliable cue for oil bees.

## Materials and Methods

### Bee study species

The oil bee *Macropis fulvipes* (Fab.) (Melittidae, Melittinae) is distributed in Europe and, like other *Macropis* species, is specialized on the oil secreting flowers of *Lysimachia* species (Primulaceae)^15^,^56^. Fatty floral oils and pollen of these plants are the only food collected by adult females for the offspring. Adult males and females feed on pollen of *Lysimachia* and females use the oil to line the brood cells^15^,^19^. Individuals used for behavioural tests were from a flight cage population^19^ (see below) and *Lysimachia*-naive, while those used for electrophysiological measurements (see below) were from a natural population in the Ecological Botanical Garden of the University of Bayreuth (EBG) and likely *Lysimachia*-experienced.

*Rediviva* (Melittidae, Melittinae) oil bees are closely related to *Macropis*, occur in Southern Africa, and also collect floral oils as food for the offspring^21^. *Rediviva neliana* Cock. is widespread in the summer rainfall area^57^. Specimens for electrophysiological measurements (see below) were collected in the Witsieshoek region of the Drakensberg while visiting oil or nectar/pollen plants.

*Melitta* bees which occur in the Holarctic and in Africa are from the same subfamily as *Macropis* and *Rediviva*, i.e. Melittinae, but species do not collect floral oils. *Melitta haemorrhoidalis* (Fab.) is distributed in Europe and is specialized on pollen of *Campanula* species^56^. Specimens for electrophysiological measurements were collected from natural populations in the EBG and served as a phylogenetic control.

The non-oil honey bee, *Apis mellifera* L., originally native to Europe and Africa, now occurs throughout the world and, in contrast to the other bee species used, belongs to the Apidae. It is among the most generalist bees and therefore is expected to have the capacity to detect a large array of scent compounds. Individuals used for electroantennographic measurements were collected in the EBG from established hives.

### Plant material and volatile collection

Floral scents for chemical analyses were collected from 58 plant species (50 oil and 8 non-oil) from different geographic regions and phylogenetically disparate plant families and genera (supplementary data, Table S1). Samples of four of these oil species were additionally used for electrophysiological analyses, and samples of *L. punctata* were additionally used for bioassays. Samples were either collected from plants growing in the natural habitat or from material collected in different greenhouses (supplementary data, Table S1). Flowers were removed from the plants using clean forceps and extracted for one minute in 2–3 ml pentane (p.a., 99%, Grüssing, Germany). The 106 obtained samples were subsequently filtered with silanized glass wool (Supelco) to remove particles and concentrated by evaporation under a gentle stream of nitrogen to a volume of 0.5 ml. The solvent extracts of leaves were used as negative controls.

### Gas Chromatography with Electroantennographic Detection (GC-EAD)

Both sexes of *M. fulvipes* were used because we did not want to use too many females from the small populations of *Macropis* and did not find differences in antennal responses between sexes in previous analyses^26^. Such potential differences were not expected to occur as both sexes visit *Lysimachia* flowers. Similar to females, males feed on pollen of the flowers after hatching, and throughout their life search for females on the flowers^19^,^20^. Antennae were tested using scent samples of four different oil species from three different plant orders (Ericales, Lamiales, Asparagales) and two different continents (Europe, Africa). By using this approach, compounds could be identified that are widespread among oil plants (phylogenetically independent) and potentially important in the oil flower/oil bee pollination system. Five *Lysimachia punctata* flower extracts (from different plants) were tested on antennae of 7 male and 6 female bees (one antenna per bee). Additionally, one flower extract of *L. congestiflora* Hemsl. and one of *Diascia integerrima* E.Mey. ex Benth. were tested on the antennae from two different males (one antenna per bee), and the flower extract of *Corycium dracomontanum* Parkman & Schelpe was tested on one male antenna.

### Electroantennography (EAG)

For the EAG tests we used five antennae from *M. fulvipes* (all female), six antennae from *R. neliana* (five males, one female), five antennae from *Melitta haemorrhoidalis* (all female), and nine antennae from honey bee workers as described above to measure dose-response curves for diacetin (diluted in acetone to four concentrations, 10^−2^, 10^−3^, 10^−4^, and 10^−5^; v/v). Antennae of *M. haemorrhoidalis* were only tested on the two highest concentrations. Both female and male antennae of *Rediviva* were used, because we found in GC-EAD analyses (unpublished data) that both sexes responded similarly to diacetin.

As a positive control we used linalool (10^−2^ in acetone), a compound widespread among plants pollinated by bees^32^, and as negative control we used acetone.

To test whether different bee species responded differently to the dilution series of diacetin, data were analysed using a repeated measurement ANOVA (STATISTICA v. 7.1.; www.statsoft.com) with individual bees as subject for repeated measures and the different *dilutions* and *bee species* as categorical factors. Tukey was used as post hoc test. Responses of *Melitta* were excluded from these analyses as only two of the four diacetin dilutions were tested in this species. Instead, we tested for a *dilution* effect in *Melitta* using a paired t-test (STATISTICA). A paired t-test was also used to test for differences in responses to acetone and the 10^−2^ dilution of diacetin in each species. For more detailed information, see Supplemental Experimental Procedures.

### Chemical Analyses

To identify the EAD-active compounds in the four species used for GC-EAD measurements, 1 μl of the flower extracts was analysed on a Varian Saturn 2000 mass spectrometer coupled to a Varian 3800 gas chromatograph fitted with a 1079 injector (Varian Inc., Palo Alto, CA, USA). Additionally, we analysed samples of the 50 oil and eight non-oil species available by GC-MS for the presence of the EAD-active compounds (for further information, see Supplemental Experimental Procedures).

### Preparation of synthetic scent mixture

For testing the attractiveness of complete and partially depleted synthetic mixtures of EAD-active substances from *L. punctata* to *M. fulvipes* bees, we prepared dilutions of the synthetic substances in acetone (99.9%, AnalaR NORMAPUR, VWR): diacetin (after purification, see Supplemental Experimental Procedures), geranic acid (98%, ABCR), heptanoic acid (99%, Aldrich), (*E*)-2-dodecenal (93%, Aldrich) and 2-tridecanone (98%, ABCR). Though EAD-active, we didn’t include 1-hydroxy-1-phenyl-2-propanone in our behavioural experiments because it failed to attract bees in previous tests^26^.

The absolute amount of synthetic compounds in the 10 μl extract offered to the bees during the bioassay was equivalent to the quantity of compounds found in extracts of 100 flowers (few flowering stems) of *L. punctata* (2 μg heptanoic acid, 4 μg geranic acid, 2 μg (*E*)-2-dodecenal, 4 μg 2-tridecanone, and 0.3 μg diacetin).

### Bioassays

Behavioural assays were needed in this study, because electroantennographically active substances do not necessarily elicit behavioural responses in insects^58^. Two-choice bioassays in the flight cage tested the importance of EAD-active floral volatiles of *L. punctata* for host plant location by *Lysimachia*-naïve *M. fulvipes* females. *Lysimachia*-naïve bees were used to study the innate basis of host plant location. Naïve bees were not trained before the experiments, did not know the test scenario before the testing started, and were not rewarded when responding (see also Supplemental Experimental Procedures). Diacetin was tested against an acetone negative control and against a natural flower extract of *L. punctata* (positive control; from 100 flowers). We further tested a natural extract against the completely synthetic (5 EAD-active compounds) mixture, as well as the complete synthetic mixture against incomplete synthetic mixtures from which one of the components was omitted. To obtain a mixture without diacetin, we additionally had to eliminate geranic acid as GC-MS analyses revealed trace amounts of diacetin (0.24 ng in 4 μg geranic acid) as a contaminant in synthetic geranic acid.

## Additional Information

**How to cite this article**: Schäffler, I. *et al.* Diacetin, a reliable cue and private communication channel in a specialized pollination system. *Sci. Rep.* **5**, 12779; doi: 10.1038/srep12779 (2015).

## Supplementary Material

Supplementary Information

Supplementary Movie

## Figures and Tables

**Table 1:** Occurrence of EAD-active substances (see Fig. 1) in oil and non-oil plants of different families/genera and floristic regions.

	Holarctic	Afrotemp.	Neotropics	Palaeotropics
*Number of taxa studied in floristic regions*				
families	1	3	6	1
genera	1	7	13	1
oil/non-oil species	7/7	18/0	22/0	3/1
*Percentage of oil/non-oil species with a specific EAD-active compound*				
diacetin	100/14	100/–	73/–	0/0
2-tridecanone	100/0	72/–	55/–	100/100
heptanoic acid	71/0	55/–	23/–	0/0
4-hydroxy-3-methoxystyrene	29/0	50/–	18/–	100/0
unkown (RI 1264)	0/0	17/–	0/–	66/0
geranic acid	29/0	0/–	2/–	0/0
(*E*)-2-dodecenal	43/0	0/–	0/–	0/0
1-hydroxy-1-phenyl-2-propanone	29/0	0/–	0/–	0/0
3,5-dimethoxytoluene	0/0	11/–	0/–	0/0

Afrotemp.: Afrotemperate region. (For detailed information see Table S1).
