# Supplementary material for: Diacetin, a reliable cue and private communication channel in a specialized pollination system
Source: Sci Rep. 2015 Aug 6;5:12779. doi: 10.1038/srep12779 (PMC4526864; doi:10.1038/srep12779)
Supplement: Supplementary Information [file srep12779-s1.doc]

**Supplementary information**

**Diacetin, a reliable cue and private communication channel in a specialized pollination system**

Short title: Signalling between oil flowers and oil bees

Irmgard Schäffler, Kim E. Steiner, Mark Haid, Sander S. van Berkel, Günter Gerlach, Steven D. Johnson, Ludger Wessjohann, and Stefan Dötterl

**Table S1:** **Oil and non-oil secreting species of the different floral regions used for chemical analyses.** The number of samples collected per species and those with diacetin is also given. Samples were either collected in Germany (BT: Bayreuth, Heckl. Hecklingen, M: Munich) or in South Africa (SAf). To obtain a single sample, in the mean 80 flowers (Min-Max: 1-500) and 277 flowers (Min-Max: 9-880) were pooled for oil and non-oil species, respectively. Afrotemp.: Afrotemperate region

| **Floristic region**  ***Plant family***  *Plant genus* | *Plant species* | Oil (O) Non-oil (N) | Nr. of samples collected/Nr. of samples with diacetin | Collected in |
| --- | --- | --- | --- | --- |
|
|  |
| **Holarctic** |  |  |  |  |
| ***Primulaceae*** | |  |  |  |
| *Lysimachia* L. | |  |  |  |
|  | *L. ciliata* L. | O | 3/3 | BT |
|  | *L. christinae* Hance | O | 3/3 | BT |
|  | *L. congestiflora* Hemsl. | O | 3/3 | BT |
|  | *L. nummularia* L. | O | 3/3 | BT |
|  | *L. punctata* L. | O | 3/3 | BT |
|  | *L. vulgaris* L. | O | 5/5 | BT |
|  | *L.patungensis* Hand.-Mazz. | O | 2/2 | BT |
|  | *L. arvensis* L. | N | 1/0 | BT |
|  | *L. atropurpurea*L. | N | 1/0 | BT |
|  | *L. clethroides*Duby | N | 3/0 | BT |
|  | *L. ephemerum* L. | N | 2/0 | BT |
|  | *L. nemorum* L. | N | 3/0 | BT |
|  | *L. maritima* (L.) Galasso, Banfi & Soldano | N | 1/0 | Heckl. |
|  | *L. minoricensis* J.J.Rodr. | N | 1/0 | BT |
|  | *L. thyrsiflora* L. | N | 5/1 | BT |
| Afrotemp. |  |  |  |  |
| ***Scrophulariaceae*** | |  |  |  |
| *Diascia* Link&Otto | |  |  |  |
|  | *D. capensis* Britten | O | 1/1 | SAf |
|  | *D. cordata* N.E.Br. | O | 1/1 | SAf |
|  | *D. barbarae* Hook. f. | O | 4/4 | BT |
|  | *D. integerrima* E.Mey. ex Benth. | O | 1/1 | SAf |
|  | *D. purpurea* N.E.Br. | O | 3/3 | SAf |
|  | *D. vigilis* Hilliard & B.L.Burtt | O | 4/4 | SAf |
| *Hemimeris* L. | |  |  |  |
|  | *H. racemosa* (Houtt.) Merr. | O | 1/1 | SAf |
| ***Orchidaceae*** | |  |  |  |
| *Corycium* Sw. | |  |  |  |
|  | *C. dracomontanum* Parkman & Schelpe | O | 2/2 | SAf |
|  | *C. orobanchoides* (L.f.) Sw. | O | 1/1 | SAf |
|  | *C. nigrescens* Sond. | O | 1/1 | SAf |
| *Pterygodium* Sw. | |  |  |  |
|  | *P. catholicum* (L.) Sw. | O | 1/1 | SAf |
|  | *P. magnum* Rchb. f. | O | 1/1 | SAf |
| *Disperis* Sw. | |  |  |  |
|  | *D. fanninae* Harv. | O | 1/1 | SAf |
|  | *D. oxyglossa* Bolus | O | 1/1 | SAf |
|  | *D. villosa* (L.f.) Sw. | O | 1/1 | SAf |
| *Huttonaea* Harv. | |  |  |  |
|  | *H. grandiflora* (Schltr.) Rolfe | O | 1/1 | SAf |
|  | *H. pulchra* Harv. | O | 1/1 | SAf |
| ***Stilbaceae*** | |  |  |  |
| *Bowkeria* Harv. | |  |  |  |
|  | *B. sp.* | O | 1/1 | SAf |
| **Neotropics** |  |  |  |  |
| ***Orchidaceae*** | |  |  |  |
| *Oncidium* Sw. | |  |  |  |
|  | *O. sotoanum*  R. Jiménez & Hágsater | O | 2/2 | M/BT |
| *Sigmatostalix* Rchb.f. | |  |  |  |
|  | *S. cuculligera (*Schltr.) Garay | O | 1/1 | M |
|  | *S. guatemalensis* Schltr. | O | 1/1 | M |
|  | *S. radicans* Rchb.f. | O | 1/1 | BT |
| *Dipteranthus* Barb. Rodr. | |  |  |  |
|  | *D. obliquus*  (Schnee) Garay & Dunst. | O | 1/1 | M |
| ***Iridaceae*** | |  |  |  |
| *Trimezia* Salisb. ex Herb. | |  |  |  |
|  | *T. sincorana* Ravenna | O | 1/1 | M |
| *Ennealophus* N.E.Br. | |  |  |  |
|  | *E. euryandrus* (Griseb.) Ravenna | O | 1/1 | M |
| ***Calceolariaceae*** | |  |  |  |
| *Calceolaria* L. | |  |  |  |
|  | *C. elatior* Griseb. | O | 1/1 | M |
|  | *C. integrifolia* L. | O | 1/1 | M |
|  | *C. sp* | O | 1/1 | BT |
| ***Malpighiaceae*** | |  |  |  |
| *Bunchosia* Rich. ex Juss. | |  |  |  |
|  | *B. spec.* | O | 2/0 | M |
| *Gaudichaudia* Kunth | |  |  |  |
|  | *G. mucronata* (DC.) A. Juss | O | 1/1 | M |
| *Heteropteris* Kunth | |  |  |  |
|  | *H. chrysophylla* (Lam.) Kunth | O | 1/1 | M |
| *Malpighia* L. | |  |  |  |
|  | *M. coccigera* L. | O | 1/1 | BT |
|  | *M. fucata* Ker Gawl. | O | 1/0 | M |
|  | *M. glabra var. undulata* Nied. | O | 1/0 | M |
|  | *M. urens* L. | O | 2/2 | M |
| *Stigmaphyllon* A. Juss. | |  |  |  |
|  | *S. ciliatum* (Lam.) A. Juss. | O | 2/2 | M |
|  | *S. sinuatum* (DC.) A. Juss. | O | 1/0 | M |
| ***Solanaceae*** | |  |  |  |
| *Nierembergia* Ruiz & Pav. | |  |  |  |
|  | *N. scoparia* Sendtn. | O | 1/0 | M |
|  | *N. hippomanica* Miers | O | 4/0 | BT |
| ***Plantaginaceae*** | |  |  |  |
| *Angelonia* Humb. & Bonpl. | |  |  |  |
|  | *A. angustifolia* Benth. | O | 1/1 | BT |
| **Palaeotropics** |  |  |  |  |
| ***Cucurbitaceae*** | |  |  |  |
| *Momordica* L. | |  |  |  |
|  | *M. cissioides* Planch.ex Benth male | O | 1/0 | M |
|  | *M. foetida* Schumach. male | O | 1/0 | M |
|  | *M. boivinii* Blume female | O | 2/0 | BT |
|  | *M. charanthia* L. male flowers | N | 1/0 | BT |
|  | *M. charanthia* L. female flowers | N | 1/0 | BT |

**Table S2:** **Overall floral oil chemistry**. Presence of diacetin in plant genera from which species were included in our analyses. Diacetin was expected to occur in plants that have oils with a glycerol moiety and an acetyl group, and to not occur in plants having other oils. Unexpected results are printed in bold. Afrotemp.: Afrotemperate region

| floristic region | genus^references^ | oil chemistry | | diacetin expected in the samples | detected diacetin in our samples |
| --- | --- | --- | --- | --- | --- |
|  |  | presence of | |  |  |
|  |  | glycerol moiety | acetyl group |  |  |
| Holarctic | *Lysimachia* ^1, 2, 3, 5^ | yes | yes | yes | yes |
| Afrotemp. | *Diascia* ^1, 4, 5^ | yes | yes | yes | yes |
|  | *Trimezia* ^1^ | yes | yes | yes | yes |
|  | *Corycium* ^5^ | yes | yes | yes | yes |
|  | *Pterygodium* ^5^ | yes | yes | yes | yes |
| Neotropics | *Angelonia* ^1, 5^ | yes | yes | yes | yes |
|  | *Nierembergia* ^1, 6^ | no | yes | no | no |
|  | *Malpighia* ^1, 5^ | yes | yes | yes | yes |
|  | *Stigmaphyllon* ^5^ | yes | yes | yes | yes |
|  | *Bunchosia* ^5^ | yes | yes | **yes** | **no** |
|  | *Sigmatostalix* ^1^ | yes | yes | yes | yes |
|  | *Calceolaria* ^7^ | yes | yes | yes | yes |
|  | *Oncidium* ^8, 5^ | yes | yes | yes | yes |
| Palaeotropics | *Momordica* ^5, 9^ | yes ^9^ | yes ^5^ | **yes** | **no** |

^1-9^: References

1. Seipold, L. *Blütenöle - Chemische Analyse, Biosynthese Und Betrachtungen Zur Entstehung Von Ölblumen*. (PHD Dissertation. Mathematisch-Naturwissenschaftlich-Technische Fakultät. Martin-Luther-Universität Halle-Wittenberg, 2004).

2. Cane, J.H. Foraging, grooming, and mating behaviors of *Macropis nuda* (Hymenoptera: Melittidae) and use of *Lysimachia ciliata* (Primulaceae) oils in larval provisions and cell lining. *Am. Midl. Nat.* **110,** 257-264 (1983).

3. Vogel, S. *Ölblumen Und Ölsammelnde Bienen, Zweite Folge:* Lysimachia *und* Macropis. Vol. 54 (Akademie der Wissenschaft und der Literatur, Franz Steiner Verlag Wiesbaden GmbH, 1986).

4. Dumri, K., Seipold, L., Schmidt, J., Gerlach, G., Dötterl, S., Ellis, A.G. *et al.* Non-volatile floral oils of *Diascia* spp. (Scrophulariaceae). *Phytochemistry* **69,** 1372-1383 (2008).

5. Dumri, K. *Chemical Analyses Of Non-Volatile Flower Oils And Related Bee Nest Cell Linings*. (PHD Dissertation. Mathematisch-Naturwissenschaftlich-Technische Fakultät, Martin-Luther-Universität Halle-Wittenberg, Halle, 2008).

6. Simpson, B.B. & Neff, J.L. Floral rewards: Alternatives to pollen and nectar. *Ann. Mo. Bot. Gard.* **68,** 301-322 (1981).

7. Vogel, S. *Ölblumen Und Ölsammelnde Bienen*. Vol. 7 (Akademie der Wissenschaft und der Literatur, Franz Steiner Verlag Wiesbaden GmbH, 1974).

8. Reis, M.G., Faria, A.D., Bittrich, V., Amaral, M.D.C.E. & Marsaioli, A.J. The chemistry of flower rewards - *Oncidium* (Orchidaceae). *J. Braz. Chem. Soc.* **11,** 600-608 (2000).

9. Vogel, S. *Ölblumen Und Ölsammelnde Bienen, Dritte Folge:* Momordica*,* Thladianthia *und die* Ctenoplectridae. Vol. 73 (Akademie der Wissenschaft und der Literatur Franz Steiner Verlag Wiesbaden GmbH, 1990).

**Supplementary Experimental Procedures**

**Gas Chromatography with Electroantennographic Detection (GC-EAD).**

Electrophysiological experiments were performed with flower samples on a gas chromatograph (HP 5890 series 2) equipped with a flame ionization detector (FID) and an EAD setup (temperature controller TC-02, two-channel universal serial bus acquisition controller IDAC-2 and stimulus controller CS-01) provided by Syntech (Hilversum, Netherlands)^1^. ^111^The gas chromatograph was equipped with a ChromatoProbe kit (CPAV6890, Aviv Analytical LTD, Hod Hasharon, Israel) allowing the analysis of “dirty” samples ^2^, and a ZB-5 column (length 30 m, inner diameter 0.32 mm, film thickness 0.25µm, Phenomenex). The ChromatoProbe kit was used for analyses of pentane floral extracts, which not only contained volatiles but also “non-volatile” floral oils. One micro liter of a flower sample in a small vial was placed into the injector port by means of the ChromatoProbe (injector temperature: 260°). Compounds not vaporized remained in the sample vial which was discarded after use. The samples were injected splitless at an oven temperature of 40°C, followed by opening the split vent after 1 min and heating the oven at a rate of 10°C/min to 260°C. The end temperature was held for 5 min. The column was split at the end by the four arm flow splitter GRAPHPACK 3D/2 (Gerstel, Mühlheim, Germany) into two pieces of deactivated capillary (length 50 cm, ID 0.32 mm) leading to the FID and EAD-setup, respectively. Makeup gas (He, 16ml/min) was introduced through the fourth arm of the splitter. For measurements, the insect antenna was cut at the base and tip, and mounted between glass micropipette electrodes filled with insect ringer (8.0 g/l NaCl, 0.4 g/l KCl, 0.4 g/l CaCl_2_). The electrodes were connected to silver wires.

**Electroantennography (EAG).**

The general EAG protocol followed Jhumur et al. ^3^. Antennae were stimulated at 2 min intervals in using the following sequence: acetone, linalool, different diacetin concentrations (lowest to highest), linalool, and acetone.

For every stimulus, 20 µl of the test solution was applied on filter paper (0.3 x 5cm). The solvent was allowed to evaporate before the strip was placed in a glass pasteur pipette (15 cm in length). Stimuli were released into a continuous flow of humidified air passing over the antenna with pulse duration of 0.5 sec, and a flow of 10 ml/sec regulated by the Syntech CS-01 Stimulus Controller (Bayreuth lab, Germany; antennae of *Macropis*, *Melitta*, and *Apis*) or the Syntech CS-55 Stimulus Controller (Pietermaritzburg lab, South Africa; antennae of *Rediviva neliana*). Data were analysed using the software EAGPro 1.0 provided by Syntech. To compensate for the loss of antennal sensitivity during the measurements, antennal responses to linalool were used to normalize the antennal responses towards acetone and diacetin (“normalize data” option in EAGPro). Thereafter, the normalized linalool response was set as 100%, and the responses to the different diacetin dilutions as well as to acetone were given in relation to that response.

**Chemical Analyses.**

For analysis, a sample was placed in a quartz vial and inserted in the injector by using the ChromatoProbe kit of Varian ^4, 5^. The injector split vent was opened and the injector heated to 40°C to flush any air from the system. After 2 min, the split was closed and the injector heated with a rate of 200°C/min to 260°C, then held at this temperature for 2 min, after which the split vent opened and the injector cooled down. A ZB-5 column (5% phenyl polysiloxane) was used for the separation (60 m long, inner diameter 0.25 mm, film thickness 0.25 µm, Phenomenex). Helium carrier gas flow was 1.0 ml/min. GC oven temperature was held for 4.5 min at 40°C, then increased by 6°C/min to 300°C and held at this end temperature for 15 min. The MS-interface temperature was 290°C and the ion trap worked at 175°C. The mass spectra were taken at 70 eV (in EI mode) with a scanning speed of 1 scan/s from m/z 30 to 650. The GC-MS data were processed using the Saturn Software package 5.2.1.

Component identification was carried out using the NIST 08 mass spectral database or MassFinder 3, and confirmed by comparison with retention times of authentic standards. Retention time and mass spectra of diacetin isomers were compared with data provided by Nebel ^6^. In all samples analysed, diacetin occurred as the 1,2- and 1,3-isomers, but in *L. vulgaris*, we also detected both enantiomers of 1,2-diacetin (Schäffler and Dötterl, unpublished data). In the present study, we did not discriminate between the different isomers, because our sample, with similar amounts of 1,2- (racemate) and 1,3-isomers,was sufficient to elicit the maximum behavioural response in *Macropis* bees (see Results). If different isomeric mixtures were differentially discriminated by different species of oil bees, the communication channel could be even more intimate. However, there is no indication of such extreme levels of specialization in this pollination system, because multiple oil plant species often attract the same bee species and a specific bee species often visits several/all of the oil plant species available in a specific habitat ^7^.

To quantify the absolute amount of the emitted EAD-active compounds, we added 1 µg of
3‑chloro‑4‑methoxytoluene (used as an internal standard) in five *L. punctata* flower extract samples.

**Purification of diacetin.** Technical diacetin (technical grade 50%, ABCR) contained glycerin, mono- and triacetin. To separate diacetin from the other substances, the mixture was chromatographed on a silica gel column (Merck, Silica gel 60, 63 – 200 μm) and eluted with EtOAc/*n*-hexane (4:1, v/v). Fractions containing diacetin (2 g) were evaporated to dryness and re-dissolved in 3 mL CH_3_CN. Further purification was obtained by preparative HPLC (Merck- L-7150 LaChrom pump, Hitachi L-7400 UV Detector, Degasser, Hitachi D-2500 Chromato Integrator). Isocratic chromatography was carried out with CH_3_CN/H_2_O (9:1, v/v) on a YMC-Pack column (ODS-A, 150x20 mm, 5 µm particle size) at a flow rate of 10 mL min^-1^. 1,2- and 1,3-Diacetin eluted between 5 and 8 minutes. Purification was monitored by ESI-MS (Applied Biosystems API-150EX) and ^1^H- NMR-Spectroscopy (Varian NMRS 600).

**Bioassays.** A *Macropis fulvipes* population was established in a flight cage (7.2 x 3.6 x 2.2 m; wood-framed mesh gauze placed in a greenhouse at the EBG ^8^. Each bee was individually marked with a plastic disc (Opalith number plates, 1-99, in five colours), commonly used for marking honey bee queens, after emerging. We did not offer the bees *Lysimachia* or other oil host plants and bees used for behavioural experiments were *Lysimachia*-naïve with respect to oil and pollen foraging. However, we offered the bees flowering *Geranium sanguineum* L. and *Origanum vulgare* L. as nectar sources and they were additionally provided with a sugar solution (30%, a 1:1 v:v mixture of glucose and fructose) that was added to the *Geranium* flowers or to an artificial feeder ^9^ . In the field, *M. fulvipes* visits Geraniaceae and Lamiaceae, among other families, as nectar source for its own energy supply ^10^.

All experiments were conducted in June 2010 on sunny days during 9 a.m. and 5 p.m., when flight activity of bees was high. Test substances were offered on the bottom of a cold and inverted glass beaker (Schott DURAN), cleaned with pentane and ethanol, and heated for 2 hours at 250°C before the experiment. Bees that approached within 5 cm of a sample were caught with an insect net and stored on ice until the test was finished. Thereby we prevented that a single bee specimen was counted more than once (pseudo replication) at a specific bioassay and that collected bees heated up too much. The bioassays were stopped when bee approaches ceased for at least 5 minutes, otherwise they lasted for a maximum of 20 minutes. The relative position of the control/treatments was randomized. This presentation method was modified when testing synthetic samples, because bees typically responded very quickly and only for 1-3 min at the beginning of the experiment. To obtain sufficient data for statistical analyses, two of the attractiveness tests were repeated, once with diacetin against a negative control and once with the natural extract against a synthetic mixture. Individuals responding in both replicates were counted only once (first replicate). The positioning of the samples was randomized.

The number of bees available for bioassays was limited in this non-model organism and the opportunities to conduct bioassays with *Lysimachia*-inexperienced individuals were constrained as it is difficult to obtain large numbers of bees in the flight cage and inexperienced bees are only available for 1-2 weeks per year, respectively. The dependency on warm and sunny days further constrains the number of suitable days available for performing bioassays in a season ^11^.

Exact binomial tests were used to test whether the samples tested against each other attracted significantly different numbers of bees. The spread sheet provided by http://udel.edu/~mcdonald/statfishers.html ^12^ was used for calculations. A Fisher’s exact test (2 x 6 contingency table; http://www.physics.csbsju.edu/stats/exact_NROW_NCOLUMN_form.html) was calculated to test whether bees responded differently to samples that were tested against the complete synthetic mixture.

**Supplemental References**

1 Dötterl, S., Füssel, U., Jürgens, A. & Aas, G. 1,4-Dimethoxybenzene, a floral scent compound in willows that attracts an oligolectic bee. *J. Chem. Ecol.* **31,** 2993-2998 (2005).

2. Amirav, A., Jing, H., Gordin, A., Poliak, M. & Dagan, S. ChromatoProbe and SnifProbe sample introduction devices for Mass Spectrometry sampling and GC and GC-MS analysis. http://www.tau.ac.il/chemistry/amirav/dsi.shtml (2011).

3. Jhumur, U., Dötterl, S. & Jürgens, A. Floral odors of *Silene otites*: their variability and attractiveness to mosquitoes. *J. Chem. Ecol.* **34,** 14-25 (2008).

4. Amirav, A. & Dagan, S. A direct sample introduction device for mass spectrometry studies and gas chromatography mass spectrometry analyses. Eur. Mass Spectrom. **3,** 105-111 (1997).

5. Dötterl, S. & Jürgens, A. Spatial fragrance patterns in flowers of *Silene latifolia*: Lilac compounds as olfactory nectar guides? *Plant Syst. Evol.* **255,** 99-109 (2005).

6. Nebel, B., Mittelbach, M. & Uray, G. Determination of the composition of acetylglycerol mixtures by 1H NMR followed by GC investigation. *Anal. Chem.*, **80,** 8712-8716 (2008).

7. Schäffler, I., Balao, F. & Dötterl, S. Floral and vegetative cues in oil-secreting and non-oil-secreting *Lysimachia* species. *Ann. Bot.* **110**, 125-138, (2012).

8. Dötterl, S. & Schäffler, I. Flower scent of oil-producing *Lysimachia punctata* as attractant for the oil-bee *Macropis fulvipes*. *J. Chem. Ecol.* **33,** 441-445 (2007).

9. Dötterl, S., Milchreit, K. & Schäffler, I. Behavioural plasticity and sex differences in host finding of a specialized bee species. *J. Comp. Physiol. A* **197**, 1119-1126 (2011).

10. Vogel, S. *Ölblumen Und Ölsammelnde Bienen, Zweite Folge:* Lysimachia *und* Macropis. Vol. 54 (Akademie der Wissenschaft und der Literatur, Franz Steiner Verlag Wiesbaden GmbH, 1986).

11. Bohman, B. et al. Discovery of pyrazines as pollinator sex pheromones and orchid semiochemicals: implications for the evolution of sexual deception. New Phytol. 203, 939-952 (2014).

12. McDonald, J.H. *Handbook Of Biological Statistics*. 2^nd^ ed (Sparky House Publishing Baltimore, Maryland, U.S.A, 2009).

**Supplementary Movie**

**Movie 1.** Attractive response of *Macropis fulvipes* oil-collecting bees to a synthetic mixture that contained diacetin and four other electrophysiologically active substances. The mixture was offered on the bottom of an inverted glass beaker.
